# Evaluation of Hip Preservation–related Patient Education Materials From Leading Orthopaedic Academic Centers in the United States and Description of a Novel Video Assessment Tool

**DOI:** 10.5435/JAAOSGlobal-D-20-00064

**Published:** 2020-06-03

**Authors:** Ali Parsa, Mark Nazal, Rik J. Molenaars, Ravi R. Agrawal, Scott D. Martin

**Affiliations:** From the Sports Medicine, Department of Orthopaedic Surgery, Massachusetts General Hospital, Harvard Medical School, Boston, MA (Dr. Parsa, Mr. Nazal, Dr. Molenaars, Agrawal, and Dr. Martin), and Orthopaedic Research Center, Mashhad University of Medical Sciences, Mashhad, Iran (Dr. Parsa).

## Abstract

The readability, reliability, and quality of online hip preservation–related patient education materials from the top 20 orthopaedic academic centers in the United States were evaluated.

**Methods:**

The patient educational materials were evaluated with the following assessment tools: Flesch-Kincaid (FK) readability test, Flesch Reading Ease formula, LIDA instrument, and DISCERN tool. Videos were assessed using the Patient Educational Video Assessment Tool (PEVAT), an author-developed scoring system.

**Results:**

A total of 121 educational items were reviewed. Median (interquartile range) or mean ± SD of the FK level, Flesch Reading Ease, LIDA, and DISCERN scores were 11.00 (3.00), 47.32 ± 12.14, 41.00 (6.00), and 64.00 (7.00), respectively. Higher ranking was correlated with higher FK (ρ = −0.21, *P* value = 0.034), higher DISCERN score (ρ = −0.39, *P* value < 0.005), and a lower PEVAT score (r = 0.61, *P* value = 0.034). The PEVAT score found that 83% of videos were classified as high quality.

**Discussion:**

An analysis of the hip preservation patient education text articles found low readability. Overall, high ranking was associated with poorer readability, higher quality text content, and lower quality video content. Video content was found to be predominantly of high quality. Improving the educational accessibility and effect of hip preservation–related topics may result in improved treatment outcomes.

Developing technologies have revolutionized communication between health professionals and patients, completely changing patient education. With the exponential growth in web-based, health-related information, patients can access information readily; however, concerns exist regarding the relevance, readability, and accuracy of information.^1-3^ Health literacy is a predictor of the overall patient health status, and interestingly, low health literacy has been shown to influence health outcomes toward higher hospitalization rates and poorer outcomes.^4,5,6,7,8^ Studies have shown that orthopaedic patients have a limited comprehension of both their pathologic condition and potential treatment interventions.^9-11^ Therefore, studies have recently directed focus toward evaluation and improvement of orthopaedic patient education materials.^12,13^

Academic orthopaedic centers often provide reliable sources of information for patients on their websites, making trusted information always available to patients. Studies have evaluated patient education resources from leading orthopaedic organizations^3,13,14,15,16^ and orthopaedic academic centers^17^ in isolated orthopaedic specialties and conditions; however, patient resources related to the bourgeoning specialty of hip preservation have yet to be extensively assessed. Because patient education materials have been shown to influence patient decisions and compliance,^18,19^ review of current materials is key to minimizing irrelevant or outdated information that may ultimately affect patient outcomes. Our goal was to evaluate the readability, reliability, and quality of hip preservation–related patient education materials provided by leading orthopaedic academic centers and to propose a novel method of video content assessment.

## Methods

In December 2018, we searched and reviewed the hip preservation–related patient education materials from the top 20 orthopaedic academic centers, according to the 2017 to 2018 US News and World Report orthopaedic specialty rankings.^20^ The rationale for studying the top centers was that it would provide the best scenario sampling of high quality, accurate, and complete patient education materials based on the likely resources, infrastructure, and specialization of these centers.

These centers' websites were searched independently by two physicians for hip preservation–related patient education materials, including text articles and videos, and were noted for review. The patient education materials on the websites of the centers were found via two methods. First, some orthopaedic centers had dedicated “hip preservation” website subsections, and all materials were included and reviewed from these. Second, for completion, all patient education materials within the orthopaedic departments' websites were reviewed for “hip preservation” content, regardless of distinct “hip preservation” webpages. Hip preservation education materials included were organized into the following 12 categories: general information, impingement, arthroscopy, labral tear, surgical dislocation, hip osteotomy (including hip dysplasia), rehabilitation, snapping hip, trochanteric bursitis, sports hernia, groin pain, and osteonecrosis. Only the content directed toward patient education was included, with articles, videos, or web links directed toward healthcare professionals excluded from the analyses.

### Readability—Flesch-Kincaid and Flesch Reading Ease Assessments

Readability was assessed by the Flesch-Kincaid (FK) grade and Flesch Reading Ease (FRE) formula, which have been used extensively for the determination of objective, numerical reading level.^12,13,21^ Each educational text content was copied into a Microsoft Office Word 2016 (Microsoft Corporation, Redmond, WA) document. The text was then edited to remove HTML tags as well as irrelevant text and punctuation not related to the subject. The articles were finally checked for spelling and grammar errors within Microsoft Word. This technique was originally presented by Badarudeen and Sabharwal^21^ and has consistently been used to assess the literature readability. The FK readability grade level and FRE formula calculations were done for each article using the Microsoft Word 2016 program, as previously described (Table 1). The FK grade level reports the level of academic education, via grade school level, necessary for an individual to read and comprehend the content of article, with increasing FK grade levels equating to increasing comprehension difficultly. The FRE formula generates a result from 0 to 100, with higher numbers equating to increasing ease of reading.

**Table 1:** Formulae and Interpretation of FK Level and FRE Score Readability Tests

FK level	(0.39 × mean number of words per sentence) + ([11.8 × mean number of syllables per word] − 15.59)
FRE score	206.835 − (1.015 × mean number of words per sentence) − (84.6 × mean number of syllables per word)

FRE Score	School Level (Equal to FK Level)	Notes
100.00-90.00	5th grade	Very easy to read. Easily understood by an average 11-yr-old student.
90.0-80.0	6th grade	Easy to read. Conversational English for consumers.
80.0-70.0	7th grade	Fairly easy to read.
70.0-60.0	8th and 9th grade	Plain English. Easily understood by 13- to 15-yr-old students.
60.0-50.0	10th to 12th grade	Fairly difficult to read.
50.0-30.0	College	Difficult to read.
30.0-0.0	College graduate	Very difficult to read. Best understood by university graduates.

FK = Flesch-Kincaid, FRE = Flesch Reading Ease

### Usability—LIDA Score

The LIDA instrument (Minervation Ltd, Oxford, UK) was created to evaluate the usability, accessibility, and reliability of health-related websites, with each of the three analyses scored independently for evaluation customization.^22^ Our study implemented the usability feature of the LIDA instrument to assess the following characteristics: clarity, consistency, functionality, and engagement. The articles were graded 0 to 3 (0 = never, 1 = sometimes, 2 = mostly, and 3 = always) for 18 independent questions, giving a maximum score of 54. A higher score indicated a clearer design, promoting accessibility and encouraging exploration of the website.^13,23^

### Quality—DISCERN Assessment

The DISCERN instrument was created to evaluate the reliability and quality of consumer health–related information on treatment choices.^24^ Articles were graded 0 to 5 on a three-point Likert-type scale (0 = no, 3 = partial, and 5 = yes) for 15 independent questions, giving a maximum score of 75. The final DISCERN score was then reported as a percentage of the maximum score possible. A higher score indicated a higher quality publication, conducive to concise, relevant aims and descriptive, thorough content.^25,26^

### Patient Educational Video Assessment Tool—Novel Video Assessment—Accessibility, Reliability, and Quality

Because there is a lack of validated video assessment tools,^27-29^ we created a novel health-related video assessment tool called the Patient Educational Video Assessment Test or Patient Educational Video Assessment Tool (PEVAT) to evaluate accessibility, reliability, and quality (Table 2). The tool's accessibility subscale contains 10 binary questions, each graded 0 (no) or 1 (yes) with a maximum score of 10 points. The tool's reliability subscale contains four binary questions, each graded 0 (no) or 1 (yes) with a maximum score of four points. The tool's quality subscale contains eight ternary questions, each graded 0 (no), 1 (partial), or 2 (yes) with a maximum score of 16. The three subscales are then added together to obtain a maximum video assessment score of 30.

**Table 2:** PEVAT or Patient Educational Video Assessment Tool, a Novel Quality Assessment Tool for Patient Education Videos That Evaluates Accessibility, Reliability, and Quality

Items	Points
Accessibility (1 point each):	__/10
1. Is date of video creation/publication available?	
2. Is content up to date, valuable, or contains historical information?	
3. Is video permanently accessible vs temporary or news content.	
4. Is video format versatile or supported by multiple media players/web browsers?	
5. Is the video length acceptable (<5 min)?	
6. Is the video downloadable?	
7. Are links to media sharing websites or networks (YouTube, Facebook, Flicker, etc.) available?	
8. Can viewers rate or comment on the video?	
9. Is the number of views/downloads available?	
10. Does the video have sound?	
Reliability (1 point each):	__/4
1. Does the video have a copyright/permission statement?	
2. Is the physician, healthcare provider, or source of content clear?	
3. Is the director, editor, producer, or source of technical video creation clear?	
4. Is the video free from advertisement bias?	
Quality (yes = 2, partial = 1, no = 0):	__/16
1. Is the video's intention and topic clear?	
2. Does the video uses models, animation, live bodies, etc. to explain content?	
3. Is content accurate and scientifically correct?	
4. Is the background free from visual and/or audible distractions?	
5. Is the content about a relevant medical or surgical issue?	
6. Does the video describe the aims (risks/benefits of treatment method, examination or surgical technique, etc.)?	
7. Does the video help patients, families, and/or health professionals understand a health subject or management/treatment option better?	
8. Is the physician, healthcare provider, or source of content reliable (expert in respective field)?	
Total score	__/30

### Statistical Analysis

Statistical analysis was done using R Statistical software version 3.5.2 (Foundation for Statistical Computing, Vienna, Austria). Each patient education material was classified as a either text or video modality. Then was classified as one of the following 12 topic areas within hip arthroscopy: general, impingement, arthroscopy, labral tear, surgical dislocation, hip osteotomy, rehabilitation, snapping hip, bursitis, sports hernia, groin pain, and osteonecrosis. Finally, each patient's education material was scored on the aforementioned assessment tools, as applicable: FK, FRE, LIDA, DISCERN, and PEVAT.

Study characteristics were reported as descriptive statistics, as number and percentage. Parametric statistics are reported as mean average, SD, and range, whereas nonparametric statistics are reported as median average, interquartile range (IQR), and range. Continuous variables were represented as mean average, SD, and range. Inter-rater reliability was accessed using the intraclass correlation coefficient to determine the degree of agreement between the two physician raters. Academic center ranking was the independent or predictor variable and was treated as a continuous variable. Although quantity of educational items (text articles and videos), quantity of topics covered, FK, FRE, LIDA, DISCERN, and PEVAT were the dependent or outcome variables, they were treated as continuous variables.

Normality of the continuous outcome variables were tested using the Shapiro-Wilk test to determine whether parametric or nonparametric analysis was necessary. The quantity of topics covered, FRE, and PEVAT were found to be parametric and were analyzed using the Pearson regression analysis, whereas the quantity of education items, FK, LIDA, and DISCERN were found to be nonparametric and were analyzed using the Spearman regression analysis. This allowed characterization of the correlation between academic center rank (independent/predictor variable) and each of the five assessment tools (dependent/outcome variable). In addition, the number of topics covered was also analyzed for correlation with rank and assessment tool scores. Finally, to determine whether subgroup differences existed among the top 20 centers, the centers will be subdivided into four groups, that is, ranks 1 to 5, 6 to 10, 11 to 15, and 16 to 20, and analyzed with the analysis of variance test if parametric or Kruskal-Wallis test if nonparametric; if found to be statistically significant, it was followed with a post hoc Tukey HSD test. *P* values < 0.05 were considered statistically significant.

## Results

### Study Characteristics

A total of 121 educational items, including 109 text articles (90.1%) and 12 videos (9.9%), were retrieved and evaluated by two physicians. No significant observer differences were noted in the number of articles selected or the scores calculated from text or video evaluation (intraclass correlation coefficient = 0.8). The median (IQR) quantity of educational items (text articles and videos) per center was 4.00 (3.25). The quantity of items per center spanned a range of 0 (academic ranks, 9 and 19) to 30 (academic rank, 1) (Table 3). Regression analysis between rank and number of educational items was found to be statistically significant with a moderate negative correlation of ρ = −0.53 (*P* value = 0.017).

**Table 3:** Distribution of Assessment Scores From the Top 20 US Orthopaedic Academic Centers

Academic Center Rank	Text Articles	Videos	FK Level [Median (IQR)]	FRE Score (Mean ± SD)	LIDA Score [Median (IQR)]	DISCERN Score [Median (IQR)]	PEVAT Score (Mean ± SD)
1	25	5	12.00 (2.00)	41.88 ± 10.01	41.00 (6.00)	67.00 (7.00)	**22.00 ± 2.00**
2	5	0	9.00 (1.00)	53.60 ± 7.30	*48.00 (7.00)*	65.00 (1.00)	—
3	6	1	8.00 (1.50)	59.33 ± 13.11	44.50 (5.50)	*69.00 (2.75)*	*26.00 ± NA*
4	15	0	11.00 (4.00)	45.07 ± 16.02	40.00 (3.50)	64.00 (5.50)	—
5	4	0	11.00 (2.00)	51.00 ± 3.46	38.00 (8.00)	66.00 (7.50)	—
6	2	3	—	—	—	—	23.67 ± 2.08
7	2	2	*6.50 (3.50)*	*65.00 ± 15.56*	46.50 (2.50)	68.50 (3.50)	26.50 ± 0.71
8	7	0	12.00 (0.50)	46.14 ± 1.68	41.00 (6.50)	65.00 (2.00)	—
9	0	0	—	—	—	—	—
10	6	0	11.00 (1.50)	52.00 ± 5.37	40.50 (6.25)	61.00 (6.25)	—
11	2	1	9.00 (1.00)	50.50 ± 6.36	47.00 (2.00)	66.50 (5.50)	*26.00 ± NA*
12	5	0	**13.00 (2.00)**	39.80 ± 10.35	39.00 (7.00)	62.00 (7.00)	—
13	4	0	**13.00 (2.50)**	**30.50 ± 6.61**	**37.00 (1.25)**	**58.00 (4.25)**	—
14	3	0	10.00 (2.00)	47.67 ± 11.59	43.00 (2.50)	64.00 (4.50)	—
15	4	0	11.00 (0.50)	41.00 ± 8.98	39.50 (1.50)	60.00 (1.00)	—
16	4	0	8.50 (2.00)	61.50 ± 12.23	44.50 (4.25)	64.00 (2.50)	—
17	3	0	8.00 (1.50)	59.67 ± 6.81	43.00 (1.00)	65.00 (2.00)	—
18	2	0	10.00 (1.00)	50.50 ± 3.54	41.00 (2.00)	59.50 (0.50)	—
19	0	0	—	—	—	—	—
20	10	0	10.50 (1.00)	49.60 ± 8.78	41.00 (1.75)	62.00 (3.75)	—
Total	109	12	11.00 (3.00)	47.32 ± 12.14	41.00 (6.00)	64.00 (7.00)	23.83 ± 2.44

FK = Flesch-Kincaid, FRE = Flesch Reading Ease, IQR = interquartile range, PEVAT = Patient Educational Video Assessment Tool

### Evaluation of Text Materials

#### Readability—Flesch-Kincaid and Flesch Reading Ease Assessments

**Figure 1:**
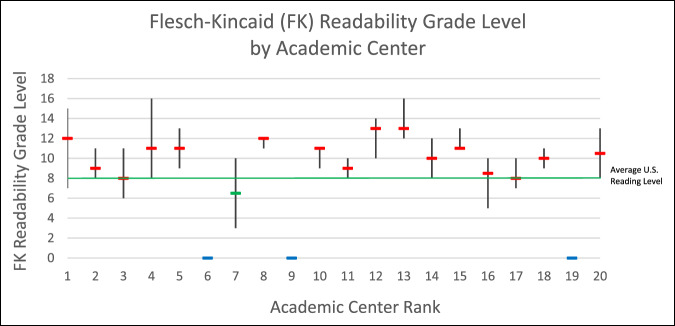
Graph demonstrating the FK readability grade levels by academic center, shown as median and range. Green line represents the average US reading level, eighth grade. FK = Flesch-Kincaid

The median (IQR) FK level of 109 text articles was 11.00 (3.00). The range consisted of articles from the 7th-ranked academic center, which was the easiest to read at a FK level = 6.50, and the articles of the 12th- and 13th-ranked academic centers were the most difficult to read at a FK level = 13.00 (Table 3 and Figure 1). In total, only 9 articles (7.4%) were at or below an eighth grade reading level, which is the average reading level in the United States (Table 4). Regression analysis between rank and FK score found a statistically significant weak negative correlation of ρ = −0.21 (*P* value = 0.034).

**Table 4:** Text Articles With Less Than or Equal to an Eighth Grade Readability Level

Academic Center Rank	Topic	FK Grade Level
7	Rehabilitation	3
16	General	5
17	General	7
16	Groin pain	8
11	Osteonecrosis	8
4	Bursitis	8
14	Osteonecrosis	8
17	Impingement	8
20	General	8

FK = Flesch-Kincaid

The mean FRE score of text articles by center was 47.32 ± 12.14. The range consisted of articles from the 7th-ranked center being the easiest to read with an FRE score of 65.00 and the 13th-ranked center's article, which was the most difficult to read with an FRE score of 30.50 (Table 3). Regression analysis between rank and FRE score was not statistically significant (r = 0.12, *P* value = 0.215).

#### Usability—LIDA Score

The median (IQR) LIDA score of text articles by center was 41.00 (1.75). The range comprising the 2nd-ranked center's website articles displayed the greatest usability with a LIDA score of 48.00 and the 13th-ranked center's website articles displayed the lowest usability with a LIDA score of 37.00. According to the LIDA score, 29 (26.6%) of all website articles demonstrated high usability (LIDA score > 44). Regression analysis between rank and LIDA score was not statistically significant. (ρ = −0.10, *P* value = 0.295).

#### Quality—DISCERN Assessment

The median (IQR) DISCERN score of text articles by center was 64.00 (7.00) or 85.33%. The range comprising the 3rd-ranked center's articles displayed the highest quality with a DISCERN score of 69.00 or 92.00% and the 13th-ranked center's articles displayed the lowest quality with a DISCERN score of 58.00 or 77.33%. Overall, 86 (78.9%) of the text articles were deemed to be at “good” quality rating or higher (DISCERN score of > 60 or 80%). Regression analysis between rank and DISCERN score found a statistically significant moderate negative correlation of ρ = −0.39 (*P* value < 0.005).

### Evaluation of Video Materials

#### Patient Educational Video Assessment Tool—Novel Video Assessment—Accessibility, Reliability, and Quality

The mean PEVAT score for the 12 videos was 23.83 ± 2.44, with a range of 22.00 to 26.50. Only 5 (25%) of the top 20 academic centers had video content available regarding hip preservation patient education. For this novel assessment, the preliminarily defined threshold of a high-quality video was >20. Among the educational videos, 10 (83.3%) deemed to be high quality. Regression analysis between rank and PEVAT score found a statistically significant strong positive correlation of r = 0.61 (*P* value = 0.034).

### Evaluation of Topic Assessment

**Figure 2:**
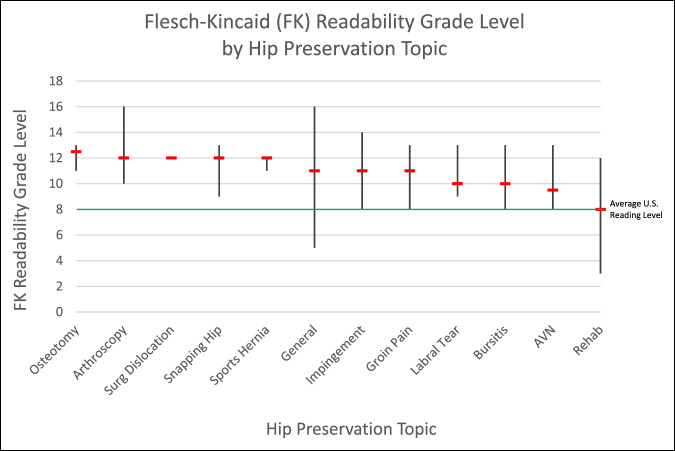
Graph demonstrating the FK readability grade levels by hip preservation topic, shown as median and range. Green line represents the average US reading level, eighth grade. FK = Flesch-Kincaid

Across the 12 topics assessed, the mean number of topics covered per center was 3.85 ± 2.64. The most common topics were general information, 34 (28.0%), and impingement, 18 (14.9%), whereas the least common topics were sports hernia, 3 (2.5%), and surgical dislocation, 1 (0.8%), (Table 5). The topics with the highest median FK readability grade were osteotomy, 12.5, followed by a 12th grade level for arthroscopy, surgical dislocation, snapping hip, and sports hernia (Figure 2). The topics with the lowest median FK readability grade were rehab, 8; osteonecrosis, 9.5; and 10th grade for both labral tear and bursitis. Finally, the number of topics covered was not significantly associated with rank (*P* = 0.153) or any of the five assessment tools (*P* > 0.05).

**Table 5:** Number of Text Articles Based on Hip Preservation Topic

Hip Preservation Topic	n (%)
General	34 (28.0)
Impingement	18 (14.9)
Bursitis	12 (10.0)
Arthroscopy	11 (9.1)
Labral tear	11 (9.1)
Rehabilitation	8 (6.6)
Osteonecrosis	8 (6.6)
Groin pain	6 (5.0)
Osteotomy	5 (4.1)
Snapping hip	4 (3.3)
Sports hernia	3 (2.5)
Surgical dislocation	1 (0.8)

### Evaluation of Subgroup Analysis

When comparing the top 20 centers as four subgroups (ranks 1 to 5, 6 to 10, 11 to 15, and 16 to 20), three statistically significance relationships were found. First, ranks 11 to 15 had a median (IQR) FK score of 11.50 (2.75) that was higher than 10.00 (2.50) for ranks 16 to 20 (*P* = 0.038). Second, ranks 11 to 15 had a mean FRE score of 40.50 ± 10.48 that was lower than 53.79 ± 9.96 for ranks 16 to 20 (*P* < 0.005). Third, ranks 1 to 5 had a median (IQR) DISCERN score of 66.00 (7.00) that was higher than 60.50 (5.50) for ranks 11 to 15 (*P* < 0.005). No statistical difference was noted in the number of educational items (articles and videos) or the number of topics between four groups.

## Discussion

Utilization of internet for patient education has greatly improved the reach of health information; however, the variability of educational resources is substantial.^1^ Orthopaedic-related patient educational materials have recently been evaluated, but little research has been focused toward the educational materials for hip preservation–related content. Our goal was to evaluate the readability, reliability, and quality of hip preservation–related patient education materials provided by leading orthopaedic academic centers and to propose a novel method of video content assessment.

Overall, the quantity and readability of the top 20 orthopaedic academic centers was highly variable. The quantity of educational items ranged from 0 to 30, with a large IQR of 3.25. Furthermore, it was found that higher ranked (lower numerical value) centers were moderately associated with a higher number of educational items (ρ = −0.53, *P* value = 0.017).

Among the 109 text articles evaluated, readability was assessed by the FK and FRE assessments, both of which found lower readability levels with substantial variability. The median FK grade level readability score was 11.00 for hip preservation–related text articles and was three grade levels above the eighth grade national average reading level in the United States^30,31^ and five grade levels above the sixth grade read level recommended by the National Institutes of Health and the American Medical Association for health-related educational information.^32^ Regression analysis found that higher ranked (lower numerical value) centers were weakly associated with a higher FK level, reflective of a lower readability (ρ = −0.21, *P* value = 0.034). Furthermore, this was supported by the subgroup analysis, in which the top 20 orthopaedic centers were divided into groups of five, finding that the centers ranked 11 to 15 had lower readability than rank 16 to 20.

Unfortunately, only 9 articles (7.4%) were at or below the eighth grade reading level, showing that hip preservation patient education material is predominantly written at a level far above the US average reading comprehension level. The only three academic centers that averaged an FK grade level readability score at or below the 8th grade level were ranked 7th (FK, 6.50), 3rd (FK, 8.00), and 17th (FK, 8.00). This finding demonstrates that the ability to present hip preservation–related health information below the eighth grade reading level is achievable but presents challenges. The health content being described is, at times, difficult to accurately convey without unavoidable medical definitions and terminology, which is why other orthopaedic subspecialties have seen similarly low readability scores.^13,33,34^ In addition, this problem is not exclusive to orthopaedics because many surgical subspecialties have uniformly low readability scores.^35-37^

Similarly, the LIDA score was used to evaluate the usability of text articles and found moderate quality with a median score of 41.00. Only 27% of the articles met the threshold for high usability. Interestingly, the LIDA score was not associated with rank, indicating that the subject matter experts at the top 20 orthopaedic centers who are producing the patient education materials are useable, regardless of the rank. Many scores were considered “fair,” which, although technically acceptable, may be concerning because the usability of patient education information could influence patient care. If a website presents information in a way that is difficult for users to find or understand, they may not return to the website for information.^23,38^ Furthermore, an additional reduction in health literacy may result because patients discontinue the use of trustworthy educational resources.

The quality of the text articles was assessed by the DISCERN score, which found favorable results. With a median of 64.00 or 85.33%, 79% of the articles found to have “good” or higher rating. Furthermore, the higher academic center rank (lower numerical value) was moderately associated with higher quality text materials (ρ = −0.39, *P* value < 0.005). Furthermore, this was supported by the subgroup analysis, finding that the centers ranked 1 to 5 had a higher quality than those ranked 11 to 15. The reasoning behind such correlation is unknown; however, higher quality publications were identified by DISCERN score as having relevant aims and thorough content. Highly ranked orthopaedic surgery centers may have more resources, infrastructure, specialization, and subject matter expertise that facilitate higher quality patient education publications. However, further research is necessary to identify areas that academic centers can improve on.

Few tools have been implemented for evaluating video content, and to the authors' knowledge, none are known to exist specifically for health-related video assessments.^27-29^ For this reason, we created a novel health-related video assessment tool to evaluate accessibility, reliability, and quality called the PEVAT (Table 2). The unique video assessment done in this study was integral in capturing the full scope of patient education material provided by academic centers. We have preliminarily defined a high quality, useful video score to be >20, but further study is necessary to validate the use and interpretation of this tool. Unfortunately, only 25% of the academic center includes video materials as part of their online patient education. However, 83% of those videos met the threshold of high quality, with direct aims and simple descriptions. This is encouraging because videos may be preferred over text materials and can be universally used by patients, regardless of the literacy or reading level^29,39^; analyses of video scores were not done because of the small number of videos retrieved and assessed. Interestingly, higher rank (lower numerical value) was negatively associated with the PEVAT score, indicating that the lower rated centers produced high-quality video materials. The reason for this association is unknown and may be due to the small sample size.

When readability was assessed by hip preservation topics, the variation in scores was similar to the analysis by academic center. The topic of “osteotomy” demonstrated the most difficult readability, whereas articles associated with “rehabilitation” showed the easiest readability. Intuitively, osteotomy-related content may present a more challenging task to explain simplistically versus rehab, which may account for the range in readability scores seen when analyzing topics. However, on average, every topic was written at a higher level than recommended, regardless of the perceived complexity.

Literature investigating web-based orthopaedic patient education materials exists, yet most studies only evaluate the readability of the information analyzed.^3,14,15,16,17,21,33,34^ Previous studies have implemented the FK, FRE, LIDA, and DISCERN scores independently for educational resource evaluation, but rarely has a comprehensive evaluation of patient education materials been done with all assessment instruments. Our study has the advantage of evaluating educational materials with all four of the aforementioned tools. In addition, studies have evaluated the patient education material from national orthopaedic organizations,^3,13,14,15,16^ from orthopaedic implant manufacturers,^40^ and from a handful of select academic centers,^17^ but our study has the benefit of evaluating the resources from the top 20 orthopaedic academic centers. This decreases selection bias, making our results more generalizable. In addition, two physician reviewers were used to procure educational materials for evaluation, which significantly limits sampling variability. Because the number of articles selected and scores from article evaluation were not deemed significantly different from each other between the two reviewers, the authors were confident that consistency was maintained throughout the study.

Although this study is the first to evaluate the readability, usability, and quality of all hip preservation–related patient education materials, the authors recognize that limitations to this study exist. The readability measures consider the number and length of words and sentence length, which has limitations because smaller words and sentences can still be difficult to understand. This is especially true with medical jargon; however, the tools used have been validated and routinely used in the literature as an effective and consistent method of evaluating readability. In addition, because the LIDA and DISCERN tools are not completely objective, variation may be seen when articles are evaluated, but consistency has still been shown when multiple observer records are done.^24,41^ Third, a selection bias of focusing exclusively on top 20 ranked orthopaedic programs exists. This bias may impair the external validity of the study to hip arthroscopy patient educational materials at other orthopaedic programs. This highlights the need for future study of additional orthopaedic programs. However, this may serve to further emphasis the effect of this study's result because even the top orthopaedic programs in the country have not achieved appropriate patient educational materials.

Recently, two studies have evaluated the readability only of arthroscopy-related topics, with one assessing hip arthroscopy readability specifically.^12,42^ Our study differs from these in that our expanded evaluation of 12 areas of hip preservation spans more than just hip arthroscopy itself. In addition, these previous studies evaluated material from internet search engines such as Google, Yahoo!, and Bing, whereas ours assessed online academic center materials. This presents the following two problems: (1) the potentially low quality and reliability of the online material and (2) the minimal direct effect one can have on content improvement.

First, the quality of the material from an internet search engine may not be as reliable or accurate as that from a vetted academic center's website because webpages can be written by anyone. Furthermore, the reliability of attempting to access information suffers because internet search engine results constantly evolve over time. In addition, results are based on one's search device, web history, geographic location, and search engine data centers, making it difficult to obtain consistent results among individuals. Second, although these studies provide valuable information regarding the materials our patients can access, the effect that healthcare providers can have on improving search engine content is severely diminished because of many factors that are implicated in their results. Conversely, the online material of academic centers can be developed by knowledgeable providers and can be a stable source of accurate information that all individuals can access independent of their device, location, etc. For this reason, it is important to not only identify the need for improving the content itself but also to establish the importance for endorsing reliable, accurate information to patients.

The most concerning problem with the content of the hip preservation–related educational materials is the low readability level found in our study. Recommendations for improvement include simpler content descriptions with condensed or smaller sentence structure. However, the addition of illustrations or video content not only makes the article easier to comprehend by giving readers an accompanying visual but also gives patients with limited literacy the opportunity to glean useful information. In this study, video content showed high quality and usability scores, further supporting this recommendation. The evaluation and improvement of academic centers' online hip preservation–related patient education materials can not only influence a patient's understanding of their condition but may also ultimately affect their clinical outcomes.

In conclusion, an analysis of hip preservation patient education text articles from the top 20 ranked orthopaedic surgery academic medical centers found low readability based on the FK and FRE assessments. A median grade level of 11.00 is substantially higher than the recommended or national average reading level. Furthermore, moderate usability and favorable quality existed. Overall, high ranking was associated with poorer readability, higher quality text content, and lower quality video content. Finally, video content was found to be predominantly of high quality. The clinical relevance of this study is seen in the direct correlation between health literacy (including readability, usability, and quality) and patient outcomes. Therefore, improving the educational accessibility and effect of hip preservation–related topics may result in improved treatment outcomes.

## References

[1] Ullrich PF Jr Vaccaro AR: Patient education on the internet: Opportunities and pitfalls. Spine 2002;27:E185-E188. 11923675 10.1097/00007632-200204010-00019

[2] Farnsworth M: Differences in perceived difficulty in print and online patient education materials. Perm J 2014;18:45-50. 25662526 10.7812/TPP/14-008 PMC4206171

[3] Eltorai AE Sharma P Wang J Daniels AH: Most American Academy of Orthopaedic Surgeons' online patient education material exceeds average patient reading level. Clin Orthop Relat Res 2015;473:1181-1186. 25475715 10.1007/s11999-014-4071-2 PMC4353543

[4] Wright JP Edwards GC Goggins K: Association of health literacy with postoperative outcomes in patients undergoing major abdominal surgery. JAMA Surg 2018;153:137-142. 28979989 10.1001/jamasurg.2017.3832 PMC5838587

[5] Hälleberg Nyman M Nilsson U Dahlberg K Jaensson M: Association between functional health literacy and postoperative recovery, health care contacts, and health-related quality of life among patients undergoing day surgery: Secondary analysis of a randomized clinical trial. JAMA Surg 2018;153:738-745. 29710226 10.1001/jamasurg.2018.0672 PMC6584305

[6] Berkman ND Sheridan SL Donahue KE Halpern DJ Crotty K: Low health literacy and health outcomes: An updated systematic review. Ann Intern Med 2011;155:97-107. 21768583 10.7326/0003-4819-155-2-201107190-00005

[7] Berkman ND Sheridan SL Donahue KE: Health literacy interventions and outcomes: An updated systematic review. Evid Rep Technol Assess (Full Rep) 2011:1-941. PMC4781058 23126607

[8] Roh YH Lee BK Park MH Noh JH Gong HS Baek GH: Effects of health literacy on treatment outcome and satisfaction in patients with mallet finger injury. J Hand Ther 2016;29:459-464. 27765527 10.1016/j.jht.2016.06.004

[9] Menendez ME Mudgal CS Jupiter JB Ring D: Health literacy in hand surgery patients: A cross-sectional survey. J Hand Surg 2015;40:798-804.e2. 10.1016/j.jhsa.2015.01.010 25746142

[10] Kadakia RJ Tsahakis JM Issar NM: Health literacy in an orthopedic trauma patient population: A cross-sectional survey of patient comprehension. J Orthop Trauma 2013;27:467-471. 23114414 10.1097/BOT.0b013e3182793338

[11] Rosenbaum AJ Tartaglione J Abousayed M: Musculoskeletal health literacy in patients with foot and ankle injuries: A cross-sectional survey of comprehension. Foot Ankle Spec 2016;9:31-36. 26123548 10.1177/1938640015593078

[12] Akinleye SD Krochak R Richardson N Garofolo G Culbertson MD Erez O: Readability of the most commonly accessed arthroscopy-related online patient education materials. Arthroscopy 2018;34:1272-1279. 29287948 10.1016/j.arthro.2017.09.043

[13] Feghhi DP Komlos D Agarwal N Sabharwal S: Quality of online pediatric orthopaedic education materials. J Bone Joint Surg Am 2014;96:e194. 25471920 10.2106/JBJS.N.00043

[14] Roberts H Zhang D Dyer GS: The readability of AAOS patient education materials: Evaluating the progress since 2008. J Bone Joint Surg Am 2016;98:e70. 27605695 10.2106/JBJS.15.00658

[15] Yi PH Ganta A Hussein KI Frank RM Jawa A: Readability of arthroscopy-related patient education materials from the American Academy of Orthopaedic Surgeons and Arthroscopy Association of North America Web sites. Arthroscopy 2013;29:1108-1112. 23726111 10.1016/j.arthro.2013.03.003

[16] Yi PH Chang MM Haughom BD Jawa A: Readability of patient education materials from the AAHS. Hand (N Y) 2014;9:393-394. 25191174 10.1007/s11552-014-9643-9 PMC4152444

[17] Ryu JH Yi PH: Readability of spine-related patient education materials from leading orthopedic academic centers. Spine 2016;41:E561-E565. 26641845 10.1097/BRS.0000000000001321

[18] Gold DT McClung B: Approaches to patient education: Emphasizing the long-term value of compliance and persistence. Am J Med 2006;119(4 suppl 1):S32-S37. 16563940 10.1016/j.amjmed.2005.12.021

[19] Majid N Lee S Plummer V: The effectiveness of orthopedic patient education in improving patient outcomes: A systematic review protocol. JBI Database System Rev Implement Rep 2015;13:122-133. 10.11124/jbisrir-2015-1950 26447013

[20] Report USNW: Best hospitals for orthopedics. 2017 https://health.usnews.com/best-hospitals/rankings/orthopedics. Accessed December 20, 2017.

[21] Badarudeen S Sabharwal S: Readability of patient education materials from the American Academy of Orthopaedic Surgeons and Pediatric Orthopaedic Society of North America Web sites. J Bone Joint Surg Am 2008;90:199-204. 18171975 10.2106/JBJS.G.00347

[22] Minervation: The LIDA instrument. 2018 http://www.minervation.com/wp-content/uploads/2011/04/Minervation-LIDA-instrument-v1-2.pdf. Accessed May 10, 2018.

[23] Eysenbach G Powell J Kuss O Sa ER: Empirical studies assessing the quality of health information for consumers on the world wide web: A systematic review. JAMA 2002;287:2691-2700. 12020305 10.1001/jama.287.20.2691

[24] Charnock D Shepperd S Needham G Gann R: DISCERN: An instrument for judging the quality of written consumer health information on treatment choices. J Epidemiol Community Health 1999;53:105-111. 10396471 10.1136/jech.53.2.105 PMC1756830

[25] Dalton DM Kelly EG Molony DC: Availability of accessible and high-quality information on the Internet for patients regarding the diagnosis and management of rotator cuff tears. J Shoulder Elbow Surg 2015;24:e135-e140. 25457189 10.1016/j.jse.2014.09.036

[26] Ellsworth B Patel H Kamath AF: Assessment of quality and content of online information about hip arthroscopy. Arthroscopy 2016;32:2082-2089. 27234649 10.1016/j.arthro.2016.03.019

[27] Pandey A Patni N Singh M Sood A Singh G: YouTube as a source of information on the H1N1 influenza pandemic. Am J Prev Med 2010;38:e1-e3. 20171526 10.1016/j.amepre.2009.11.007

[28] Matsuyama RK Lyckholm LJ Molisani A Moghanaki D: The value of an educational video before consultation with a radiation oncologist. J Cancer Educ 2013;28:306-313. 23526553 10.1007/s13187-013-0473-1 PMC4851426

[29] Abu Abed M Himmel W Vormfelde S Koschack J: Video-assisted patient education to modify behavior: A systematic review. Patient Educ Couns 2014;97:16-22. 25043785 10.1016/j.pec.2014.06.015

[30] Statistics NCfE: Basic reading skills and the literacy of the America's least literate adults: Results from the 2003 National Assessment of Adult Literacy (NAAL) supplemental studies. 2009 https://nces.ed.gov/pubs2009/2009481.pdf. Accessed May 10, 2018.

[31] Pati S Kavanagh JE Bhatt SK Wong AT Noonan K Cnaan A: Reading level of Medicaid renewal applications. Acad Pediatr 2012;12:297-301. 22682719 10.1016/j.acap.2012.04.008

[32] Institute of Medicine Committee on Health L, Nielsen-Bohlman L Panzer AM Kindig DA, eds: Health Literacy: A Prescription to End Confusion*.* Washington, DC, National Academies Press (US), 2004. 25009856

[33] Shah AK Yi PH Stein A: Readability of orthopaedic oncology-related patient education materials available on the Internet. J Am Acad Orthop Surg 2015;23:783-788. 26519430 10.5435/JAAOS-D-15-00324

[34] Bluman EM Foley RP Chiodo CP: Readability of the patient education section of the AOFAS website. Foot Ankle Int 2009;30:287-291. 19356350 10.3113/FAI.2009.0287

[35] Schmitt PJ Prestigiacomo CJ: Readability of neurosurgery-related patient education materials provided by the American Association of Neurological Surgeons and the National Library of Medicine and National Institutes of Health. World Neurosurg 2013;80:e33-e39. 22120275 10.1016/j.wneu.2011.09.007

[36] Svider PF Agarwal N Choudhry OJ: Readability assessment of online patient education materials from academic otolaryngology-head and neck surgery departments. Am J Otolaryngol 2013;34:31-35. 22959363 10.1016/j.amjoto.2012.08.001

[37] Hansberry DR Agarwal N Shah R: Analysis of the readability of patient education materials from surgical subspecialties. Laryngoscope 2014;124:405-412. 23775508 10.1002/lary.24261

[38] Health R: Proceed with caution: A report on the quality of health information on the Internet. 2001 https://www.chcf.org/wp-content/uploads/2017/12/PDF-ProceedWithCautionReportSummary.pdf. Accessed May 10, 2018.

[39] Park JS Kim MS Kim H: A randomized controlled trial of an educational video to improve quality of bowel preparation for colonoscopy. BMC Gastroenterol 2016;16:64. 27317249 10.1186/s12876-016-0476-6 PMC4912707

[40] Yi MM Yi PH Hussein KI Cross MB Della Valle CJ: Readability of patient education materials from the web sites of orthopedic implant manufacturers. J Arthroplasty 2017;32:3568-3572. 28750856 10.1016/j.arth.2017.07.003

[41] Rees CE Ford JE Sheard CE: Evaluating the reliability of DISCERN: A tool for assessing the quality of written patient information on treatment choices. Patient Educ Couns 2002;47:273-275. 12088606 10.1016/s0738-3991(01)00225-7

[42] Mehta MP Swindell HW Westermann RW Rosneck JT Lynch TS: Assessing the readability of online information about hip arthroscopy. Arthroscopy 2018;34(7):2142-2149. 29631940 10.1016/j.arthro.2018.02.039

